# Calcium Enriched Mixture and Mineral Trioxide Aggregate Activities against Enterococcus Faecalis in Presence of Dentin

**Published:** 2013-10-07

**Authors:** Hasan Razmi, Mohsen Aminsobhani, Behnam Bolhari, Farin Shamshirgar, Shadi Shahsavan, Ahmad Reza Shamshiri

**Affiliations:** a Department of Endodontics, Dental School, Dental Research Center, Tehran University of Medical Sciences, Tehran, IR Iran; b Dental School, AJA University of Medical Sciences, Tehran, IR Iran; c Private Practice, Tehran, IR Iran; d Anti-microbial Resistance Research Center, Tehran University of Medical Sciences, Tehran, IR Iran; e Dental Research Center, Dental School, Tehran University of Medical Sciences, Tehran, IR Iran

**Keywords:** Antibacterial Agent, Calcium Enriched Mixture, CEM Cement, Dentin, Enterococcus Faecalis, Mineral Trioxide Aggregate, MTA

## Abstract

**Introduction:**

The purpose of this in vitro study was to compare the antibacterial activity of Calcium Enriched Mixture (CEM) with ProRoot Mineral Trioxide Aggregate (MTA) against Enterococcus faecalis (E. faecalis) in the presence/absence of dentin powder.

**Materials and Methods:**

Two series of freshly mixed (10, 50, and 100 mg), set crushed powder (10, 50, and 100 mg), and pieces of uncrushed set (50, 100 mg) of CEM and MTA were prepared (n = 32 groups). All samples were suspended in normal saline for direct exposure test against E. faecalis; in the second series, 50 mg of the dentin powder was also added to the solution. Dentin powder suspension and bacterial suspension served as negative and positive control groups, respectively (n = 2). The suspensions were incubated at room temperature for 1, 60, and 240 min; each group was tested five times and survival of the bacteria in test solutions was assessed by 10-fold serial dilutions and cultured on Brain Heart Infusion (BHI) plates. The plates were incubated at 37ºC. The mean values of log10 CFU were calculated and compared in all tested groups. The total number of tests added up to 510 times.

**Results:**

In presence of dentin powder, freshly mixed powder from set materials, and pieces of uncrushed set materials of both tested cements killed > 95% of the bacterial cell in 1 min. Adding dentin powder caused an increase in antibacterial activity of freshly mixed powder from crushed set CEM and MTA but no acceleration in bacterial killing was observed, when dentin was mixed with set or uncrushed cements. Dentin powder alone reduced the number of viable bacteria in the 4-hour duration. There were no significant differences between different weights of freshly mixed, crushed set powder and uncrushed set of CEM cement and MTA at different times.

**Conclusion:**

Under the conditions of this in vitro study, CEM cement as well as MTA have antibacterial effects against E. faecalis. The addition of equal amounts of dentin powder to the suspension of CEM or MTA resulted in swifter elimination of bacteria.

## 1. Introduction

Microorganisms are the main factor in the development and progression of pulpal and periapical diseases as well as endodontic treatment failures (1). The outcome of endodontic treatment would depend on effective canal sealing and the ability to prevent future (re)contamination, as well as successful reduction or elimination of the associated microorganisms. Although many existing biomaterials may not provide a perfect seal, they can prevent bacterial growth (2).

Some studies have showed that calcium hydroxide (3), Bioglass (4), sodium hypochlorite 5.25%, Cetrimide 0.5% (5), combination of AH26 root canal sealer and antibiotic (6), chlorhexidine 2% (7) and Carvacrol 0.6% (8) have variable antimicrobial effects against Enterococcus faecalis (E. faecalis).

Frequently, MTA has been used as a retrograde filling material and the material of choice in cases of sealing the iatrogenic or pathologic communication pathways between the root canal system and the external root surface. Considering physical and chemical properties of MTA, it’s using as a biomaterial for root-end filling and perforation repair has been recommended in cases of failed endodontic treatments (9-11). Meanwhile several studies have been conducted to assess the antimicrobial activity of MTA (12-16). Recently, a novel endodontic cement known as Calcium Enriched Mixture (CEM) cement has been developed (17). CEM cement consists of different calcium compounds such as calcium oxide, calcium phosphate, calcium carbonate, calcium silicate and calcium hydroxide. Its physical properties are compatible to ISO 6876:2001 (18). The clinical applications of CEM are similar to those of MTA, and both cements have similar working times, pH values and dimensional stability (19). Also CEM cement has fungicidal effects against Candida Albicans even in low concentration, which is similar to MTA (20).

Recent studies have provided valuable evidence about the effects of dentin on the antimicrobial properties of endodontic disinfecting agents (21). Zhang et al. verified an increase in antibacterial effect of MTA against E. faecalis in presence of dentin (22).

Considering the lack of such evidence for the effect(s) of dentin on the antimicrobial properties of CEM cement, this in vitro study was conducted in order to evaluate the effect(s) of dentin powder on antibacterial properties of CEM cement against E. faecalis in an aqueous solution before and after setting, in comparison with MTA.

## 2. Material and Methods

### 2.1. Cements

The mineral-based endodontic cements used in this study were Calcium Enriched Mixture (BioniqueDent, Iran, Tehran) and ProRoot MTA (Dentsply/Tulsa Dental, Tulsa, OK). Three forms of freshly mixed, crushed set, and uncrushed set of the materials were used in this study. Exact amounts of 10, 50 and 100 mg of powder were mixed with instructed respective amount of liquid with a shaker (Labtron model, LS 100, Iran), for preparing the cements. In order to prepare crushed set cements, MTA and CEM were mixed with their liquid according to the manufacturers' instructions and were allowed to set in 95% relative humidity for 7 days. After complete setting, blocks of 50 and 100 mg in weight were prepared, some of which were ground into a fine powder with a particle size approximately similar to that of fresh materials (6.1 - 15 µm for MTA and 0.5 - 2.5 µm for CEM) by means of a pestle and mortar and then by the Vario-Planetary Mill (Fritsch Pulverisette 4, Idar Oberstein, Germany) and hand sieving. Size of particles were evaluated by SEM, then the powders were measured in three weights of 10, 50 and 100 mg. Totally 16 groups were prepared. All of the samples (crushed set powder, and uncrushed set) were sterilized using gamma ray in 25 kGy (Maximum dose rate, 4.09 Gy/sec, using 60 Co).

### 2.2. Preparation of the Bacterial Colonies

In this study, the test organism was E. faecalis ATCC 29212. E. faecalis were cultured in Brain Heart Infusion (BHI; Scharlau, Barcelona, Spain) for 24 h at 37°C. Bacteria were suspended in 1 mL normal saline solution with physiologic concentration (8.5%, wt/vol). The density of 4×108 CFU/mL for E. faecalis were adjusted using spectrophotometry.

### 2.3. Preparation of Dentin Powder

Intact single-rooted human teeth that were extracted due to hopeless periodontal disease were kept in sodium hypochlorite 5.25% for 30 min to remove surface soft tissues. The crown (from CEJ) and apical 3 mm of the teeth were cut off with a rotating diamond saw (Komet 925 P; Brasseler Gmbh, Lemgo, Germany) under copious water irrigation. The apical portion of the root canals were prepared using NaOCl 5.25% irrigation and K-Flex files (Mani Inc., Tochigi-Ken, Japan) up to size #45 and then were flared by Gates-Gliden drills sizes #1, 2, and 3 (Mani Inc., Tochigi-Ken, Japan). The root cementum was removed by a high speed diamond fissure bur, and the dentin of the roots were crushed with a pestle and mortar and then by the Vario-Planetary Mill and hand sieving to obtain fine dentin powder with a particle size of 0.2 - 20 µm in diameter. Particle sizes in the dentin powder were evaluated by SEM. The amount of 50 mg of the dentin powder prepared by the above mentioned method was sterilized by autoclaving at 121 °C for 15 min.

### 2.4. Evaluation the Effect of Dentin on the Antibacterial Activity of CEM and MTA

50 mg of the dentin powder was added to the micro tubes containing CEM and MTA in either freshly mixed and set stages, the latter in two forms of powder from crushed set, and blocks of uncrushed set, in different weights (totally 16 groups). Sterile dentin suspension and bacterial suspensions served as negative and positive control groups, respectively 1 mL of bacterial suspension with the concentration of 4×108 CFU/mL was added to the cements, cement/dentin powder and dentin powder suspensions, then were mixed with shaker. After incubation at room temperature for 1, 60 and 240 min, the survival of the bacteria was assessed by 10-fold serial dilutions and culture on BHI plates and incubated for 24-48 h at 37 °C. At each time interval, 50λ of the suspension (cement/ dentin powder/bacterium) was extracted from the micro-tubes. After incubation period, colonies on the plates were counted, and CFU/mL was calculated. For experimental and control groups (n = 34) the tests were performed 5 times. Consequently, the total number of tests was 510 times.

### 2.5. Data analysis

The mean values of log10 CFU with the standard deviation were calculated. Statistical analysis was performed with Statistical software SPSS version 18.0 by using Mann-Whitney test. The p-value was adjusted for the number of comparisons and finally was set at 0.0025 to prevent multiple comparison error. Kruskal-Wallis test was used to evaluate the different weights among the groups, and statistical significance was established at P < 0.05.

## 3. Results

Freshly mixed, powder from set materials, and blocks of uncrushed set of both cements killed > 95% of the bacteria in 1 min duration in presence of dentin (Table 1). It is clear that 

**Table 1.** Percent of Survived E. Faecalis after Incubation with Different Stages of CEM and MTA for 1, 60, 240 min in the Absence and Presence of Dentin Powder

Mean value	CFU (base line)		CFU (1 min)		CFU (1 h)		CFU (4 h)	
	No dentin	With dentin	No dentin	With dentin	No dentin	With dentin	No dentin	With dentin
**Freshly** **mixed MTA**	100.00 ± 0	100.00 ± 0	6.49 ± 1.07	4.16 ± 2.63	1.64 ± 1.94	0.85 ± 1.7	0 ± 0.05	0 ± 0.01
**Freshly mixed CEM**	100.00 ± 0	100.00 ± 0	6.46 ± 0.1	4.33 ± 2.38	1.23 ± 1.73	0.75 ± 1.36	0 ± 0.01	0 ± 0.01
**Set crushed MTA**	100.00 ± 0	100.00 ± 0	6.15 ± 0.81	4.10 ± 2.24	1.58 ± 1.55	1.42 ± 2.13	0.05 ± 0.17	0.01 ± 0.25
**Set crushed CEM**	100.00 ± 0	100.00 ± 0	6.23±0.9	4.08±2.37	0.7±1.34	0.61±1.23	0.02±0.07	0.01±0.05
**Set uncrushed MTA**	100.00 ± 0	100.00 ± 0	6.52 ± 0.83	4.55 ± 2.23	4.33 ± 0.81	3.56 ± 1.56	0.13 ± 0.35	0.07 ± 0.81
**Set uncrushed CEM**	100.00 ± 0	100.00 ± 0	7.06 ± 0.1	4.34 ± 2.41	3.49 ± 1.33	2.86 ± 1.66	0.04 ± 0.1	0.04 ± 0.06

**Table 2.** Mean and Standard Deviation of Log10 CFU After Incubation with Different Stages of CEM and MTA for 1, 60, 240 min

Log10 CFU						
Time			0	1 min	60 min	240 min
**Groups**	**Bacteria**		8.57 ± 0.05	8.48 ± 0.03	8.41 ± 0.06	8.38 ± 0.03
	**Dentin powder**		8.57 ± 0.05	7.4 ± 0.19	7.33 ± 0.2	7.01 ± 0.27
	**Set crushed CEM (weight)**	10 mg	8.57 ± 0.05	7.06 ± 0.35	5.43 ± 2.9	2.26 ± 2.92
		50 mg	8.57 ± 0.05	7.19 ± 0.27	1.64 ± 2.64	0 ± 0
		100 mg	8.57 ± 0.05	7.12 ± 0.3	0 ± 0	0 ± 0
	**Freshly mixed CEM (weight)**	10 mg	8.57 ± 0.05	7.23 ± 0.23	5.95 ± 2.15	0 ± 0
		50 mg	8.57 ± 0.05	7.09 ± 0.32	0 ± 0	0 ± 0
		100 mg	8.57 ± 0.05	7.15 ± 0.3	1.95 ± 3.18	0 ± 0
	**Set uncrushed CEM (weight)**	50 mg	8.57 ± 0.05	7.23 ± 0.27	7.03 ± 0.37	2.71 ± 2.86
		100 mg	8.57 ± 0.05	7.24 ± 0.24	6.26 ± 2.21	1.71 ± 2.76
	**Set crushed MTA (weight)**	10 mg	8.57 ± 0.05	7.17 ± 0.3	6.44 ± 2.27	2.9 ± 3.08
		50 mg	8.57 ± 0.05	7.16 ± 0.24	1.95 ± 3.19	0 ± 0
		100 mg	8.57 ± 0.05	7.03 ± 0.42	0 ± 0	0 ± 0
	**Freshly mixed MTA (weight)**	10 mg	8.57 ± 0.05	7.2 ± 0.25	5.21 ± 2.83	0 ± 0
		50 mg	8.57 ± 0.05	7.05 ± 0.42	0 ± 0	0 ± 0
		100 mg	8.57 ± 0.05	7.02 ± 0.46	1.44 ± 3.04	0 ± 0
	**Set uncrushed MTA (weight)**	50 mg	8.57 ± 0.05	7.19 ± 0.24	7.18 ± 0.14	1.47 ± 2.92
		100 mg	8.57 ± 0.05	7.19 ± 0.29	7 ± 0.32	1.16 ± 2.45
**Materials**	**Freshly mixed CEM**		8.57 ± 0.05	7.16 ± 0.28	2.63 ± 3.31	0.18 ± 0.97
	**Set crushed CEM**		8.57 ± 0.05	7.23 ± 0.26	6.65 ± 1.59	2.21 ± 2.78
	**Set uncrushed CEM**		8.57 ± 0.05	7.23 ± 0.25	6.65 ± 1.59	2.21 ± 2.78
	**Dentin powder**		8.57 ± 0.05	7.4 ± 0.19	7.33 ± 0.2	7.01 ± 0.27
	**Freshly mixed MTA**		8.57 ± 0.05	7.09 ± 0.38	2.22 ± 3.22	0.18 ± 1
	**Set crushed MTA**		8.57 ± 0.05	7.12 ± 0.32	2.8 ± 3.5	0.97 ± 2.21
	**Set uncrushed MTA**		8.57 ± 0.05	7.19 ± 0.26	7.09 ± 0.26	1.31 ± 2.61

dentin powder enhanced the antibacterial activity of freshly mixed and crushed set cements of both tested materials (P = 0.002). On the other hand, when dentin powder was mixed with blocks of set uncrushed cements, no acceleration in bacterial killing was observed (P = 0.15). After 1 h of exposure, freshly mixed of both CEM and MTA (50 mg) and powder from set materials (100 mg) killed all bacteria (Tables 2-3). After 4 h of exposure, freshly mixed (10, 50 and 100 mg) and crushed set powder of both cements (50 mg) killed all bacteria (Tables 2-3). The 50 mg amount of freshly mixed CEM and MTA showed quicker killing of E. faecalis at 1 h than 100 mg of these materials (P < 0.05). Freshly mixed and crushed set of both CEM and MTA (50 and 100 mg) killed more than 99% of the bacteria within 1 min (Table 2). Only small amounts of freshly mixed CEM and MTA (10 mg) killed all the bacteria during the 4 h exposure time (Tables 2-3). Fresh mixture of CEM and MTA and powder from crushed set of these materials were more effective than blocks of set uncrushed cements in killing the bacteria. 

Dentin powder reduced the number of viable bacteria during the 4 h observation. There was no statistically significant difference between various states and weights of CEM and MTA cements (P = 0.008). 

**Table 3.** Percent of Survived E. Faecalis after Incubation with Different Stages of CEM and MTA for 1, 60, 240 min

Log10 CFU						
Time			0	1 min	60 min	240 min
**Groups**	**Bacteria**		100 ± 0	77.05 ± 4.88	67.77 ± 4.56	57.5 ± 5.59
	**Dentin powder**		100 ± 0	6.87 ± 3.34	5.82 ± 2.71	2.96 ± 1.84
	**Set crushed CEM (weight)**	10 mg	100 ± 0	3.69 ± 2.41	1.81 ± 1.58	0.06 ± 0.1
			50 mg	100 ± 0	4.56 ± 2.5	0.03 ± 0.05	0 ± 0
			100 mg	100 ± 0	4 ± 2.36	0 ± 0	0 ± 0
	**Freshly mixed CEM (weight)**	10 mg	100 ± 0	4.8 ± 2.3	1.57 ± 1.56	0 ± 0
			50 mg	100 ± 0	3.89 ± 2.49	0 ± 0	0 ± 0
			100 mg	100 ± 0	4.31 ± 2.51	0.68 ± 1.47	0 ± 0
	**Set uncrushed CEM (weight)**	50 mg	100 ± 0	4.92 ± 2.55	3.34 ± 1.77	0.04 ± 0.06
			100 mg	100 ± 0	4.96 ± 2.39	2.38 ± 1.47	0.04 ± 0.07
	**Set crushed MTA (weight)**	10 mg	100 ± 0	4.49 ± 2.5	3.6 ± 2.11	0.15 ± 0.28
			50 mg	100 ± 0	4.12 ± 1.99	0.67 ± 1.41	0 ± 0
			100 mg	100 ± 0	3.69 ± 2.4	0 ± 0	0 ± 0
	**Freshly mixed MTA (weight)**	10 mg	100 ± 0	4.65 ± 2.45	1.74 ± 2.16	0 ± 0
			50 mg	100 ± 0	3.96 ± 2.79	0 ± 0	0 ± 0
			100 mg	100 ± 0	3.88 ± 2.85	0.81 ± 1.73	0 ± 0
	**Set uncrushed MTA (weight)**	50 mg	100 ± 0	4.46 ± 2.16	4 ± 1.19	0.22 ± 0.48
			100 mg	100 ± 0	4.65 ± 2.41	3.12 ± 1.81	0.04 ± 0.08
**Materials**	**Freshly mixed CEM**		100 ± 0	4.33 ± 2.38	0.75 ± 1.36	0 ± 0.01
	**Set crushed CEM**		100 ± 0	4.08 ± 2.37	0.61 ± 1.23	0.02 ± 0.07
	**Set uncrushed CEM**		100 ± 0	4.94 ± 2.41	2.86 ± 1.66	0.04 ± 0.06
	**Dentin powder**		100 ± 0	6.87 ± 3.34	5.82 ± 2.71	2.96 ± 1.84
	**Freshly mixed MTA**		100 ± 0	4.16 ± 2.63	0.85 ± 1.7	0 ± 0.01
	**Set crushed MTA**		100 ± 0	4.1 ± 2.24	1.42 ± 2.13	0.05 ± 0.17
	**Set uncrushed MTA**		100 ± 0	4.55 ± 2.23	3.56 ± 1.56	0.13 ± 0.35

## 4. Discussion

The purpose of this in vitro study was to compare the antibacterial activity of Calcium Enriched Mixture (CEM) with Mineral Trioxide Aggregate (MTA) against E. faecalis in the presence of dentin. MTA showed considerable bactericidal ability which was consistent with the results gained by Zhang et al. (22). Although Estrela et al. (23) reported that MTA did not have any antibacterial activity against E. faecalis, and Torabinejad et al. (2) detected no efficacy against E. faecalis, the results of current study are in agreement with those of Eldeniz et al. (12), Sipert et al. (14), and Zhang et al. (22) who stated that MTA either delayed or inhibited the growth of E. faecalis. However, making comparisons among studies with different methodologies seems improper.

In many studies, the antimicrobial properties of CEM have been compared with MTA. Asgary and Kamrani (24) and Zarrabi et al. (18) reported that the effective antibacterial activity of CEM against E. faecalis was superior to MTA in agar diffusion test. On the contrary, the results of this study reveal the similarity of antibacterial property of CEM with that of MTA. Those studies conducted using agar diffusion test tend to indicate chemical interaction of the tested materials with agar in the first place rather than their antibacterial effects. In this study, direct exposure test was used to assess antibacterial property of the materials. As one of the most advantageous properties of this test, reduction in the number of confounding factors and the implied effect, is worth mentioning.

E. faecalis was selected as the test organism for several reasons such as being the most frequently recovered bacteria from the unhealed cases of apical periodontitis that requiring retreatment (25), being more resistant to some of the common intracanal medicaments than other bacteria, which is believed to be due to its high alkali tolerance and the last but not least, its ability to survive during RCT (26).

So far, numerous studies investigated the effects of dentin on the antimicrobial properties of endodontic medicaments (21). The effect of dentin on the antimicrobial effectiveness of CEM has not been reported before. In this study, the dentin powder model was used to investigate the effect of dentin on the antibacterial activity of CEM and MTA. Dentin powder can serve as a simple experimental model from dentin particles produced in clinical condition during root canal preparations. The obtained particles from scraping the dentin during canal preparations are placed within smear layer which will remain in contact with intracanal medicaments. However, amount of the produced dentin powder is quantitatively less than the amount of dentin powder model. Thus, in this model the increased contact surface of the samples with bacteria and dentin powder particles, might lead to exaggerate responses which cannot be applicable to clinical conditions, and this can be assumed as the disadvantages of this model. On the other hand, when smear layer is eliminated from the root canal, the materials will be put in contact with the root dentin surface.

The present study showed that dentin facilitates the bactericidal effects of both MTA and CEM against E. faecalis. Zehnder et al. reported that dentin enhanced the antibacterial effect of bioactive glass (BAG) against E. faecalis (27). In this study BAG and dentin were pre-incubated for 24 h and the results showed a significant increase in amount of bacterial killing compared to BAG alone, which was observed from 1-5 h. Even without pre-incubation, mixing dentin with either MTA or CEM resulted in quicker killing of bacteria which was consistent with the results reported by Zhang et al. (22). Zehnder et al. (27) and Zhang et al. (22) showed that the pH values were almost kept at the same level when MTA was mixed with dentin powder. Therefore, it seems that factors other than pH value are responsible for the faster elimination of E. faecalis by the cements in the presence of dentin. Thus, it can be assumed that the increase of antibacterial property of CEM and MTA in the presence of dentin, like BAG, is due to increased silica (SiO2) dissolution. The greatest proportion in MTA after calcium oxide belongs to silica (21.20%) which is also contained in CEM (6.32%) (17). Gubler et al. suggested that the mechanism of bacterial killing by BAG- that is not directly linked to pH- is dependent on ion release from the BAG material (28). Zhang et al. stated that the increased killing of E. faecalis by BAG and MTA in the presence of dentin powder might be triggered by a mechanism similar to that suggested for BAG (22). The increased antibacterial activity may also be a result of the osmolarity changes obtained from dissolution of various mixtures of CEM and MTA, and the complex ionic flow, which take place in the interface between cements and dentin particles, and may result in an increase in antimicrobial effects of CEM and MTA. Providing a proof of these theories need further research and investigation.

Contrary to the study by Eldeniz et al., in this study, there was no significant difference between the set and freshly mixed samples, which can be attributed to the different methodologies (12). In addition, in the aforementioned study for preparation of set MTA samples, the cement was mixed 3 days prior to testing. In the present study, MTA was set during 7 days, and there was also some time interval between the sample's preparation and testing.

Zhang et al. (22) and Haapasalo et al. (29) reported that dentin powder alone did not cause any reduction in the number of viable bacteria during the experiment, while in the current study, dentin powder reduced the number of bacteria during 4 h which may be due to the use of different methods in dentin powder preparations. The racial differences and consequently the composition of used tooth dentin, may have an impact on the results. In the study by Haapasalo et al. (29) and Zhang et al. (22), the removal of pulp and root cementum was not mentioned. In addition, racial difference and contexture of the used dentin may affect the results.

In comparison with freshly mixed and crushed set powder, blocks of uncrushed set of both CEM and MTA had lower antibacterial activity. This may indicate the effect of increased contact surface in improvement of antibacterial activity. Since the set samples of CEM and MTA have more alkaline pH than the fresh ones, the set cements were used in addition to the freshly mixed CEM and MTA. To investigate the effectiveness of increasing the contact surface of CEM and MTA set cements, some of the blocks of the set cements were crushed into powder with a particle size similar to that of fresh, non-set materials. Soheilipour et al. stated that CEM contained the greatest number of particles within the range of 0.5-2.5 μm (30). Also CEM had the highest percentage within this range (25.7%), while Root MTA’s highest distribution range was between 6.1-15 μm (26.3%); for this reason, this particle sizes selected for crushed set cements.

Powder from crushed set cements was more effective than fresh mixture in killing the bacteria. It might be due to more alkaline pH of set CEM and MTA. Freshly mixed and crushed set of both CEM and MTA (50 and 100 mg), in comparison with 10 mg of these materials, killed more than 99% of the bacteria within 1 min which indicates the effect of higher amounts of cements on the antibacterial efficacies of them. After 1 h of exposure, freshly mixed of both CEM and MTA (50 mg) in the presence of dentin showed quicker killing of E. faecalis than 100 mg of these materials. Only minor amounts of freshly mixed of CEM and MTA (10 mg) killed all the bacteria during the 4 h experiment. Therefore, there is more need for further studies to test for the possible effect of the dissolved material on E. faecalis in freshly mixed and set samples in the presence of dentin powder.

## 5. Conclusion

The results of this in vitro study revealed similar antibacterial properties of CEM and MTA against E. faecalis and showed that in presence of dentin, their antibacterial properties increased. According to the results, CEM cement has clinical acceptance in endodontic treatments, especially when the antimicrobial effect against E. faecalis is required.
